# Fitting parametric random effects models in very large data sets with application to VHA national data

**DOI:** 10.1186/1471-2288-12-163

**Published:** 2012-10-24

**Authors:** Mulugeta Gebregziabher, Leonard Egede, Gregory E Gilbert, Kelly Hunt, Paul J Nietert, Patrick Mauldin

**Affiliations:** 1 Center for Disease Prevention and Health Interventions for Diverse Populations, Ralph H. Johnson Veterans Affairs Medical Center, Charleston, SC, USA; 2 Division of Biostatistics & Epidemiology, Medical University of South Carolina, 135 Cannon St, Charleston, SC, 29425, USA; 3 Center for Health Disparities Research, Division of General Internal Medicine, Medical University of South Carolina, Charleston, SC, USA; 4 Department of Clinical Pharmacy and Outcome Sciences, South Carolina College of Pharmacy, Charleston, SC, USA

**Keywords:** Generalized linear mixed model, Homogeneity, Random effect meta regression, Longitudinal data, Very large dataset

## Abstract

**Background:**

With the current focus on personalized medicine, patient/subject level inference is often of key interest in translational research. As a result, random effects models (REM) are becoming popular for patient level inference. However, for very large data sets that are characterized by large sample size, it can be difficult to fit REM using commonly available statistical software such as SAS since they require inordinate amounts of computer time and memory allocations beyond what are available preventing model convergence. For example, in a retrospective cohort study of over 800,000 Veterans with type 2 diabetes with longitudinal data over 5 years, fitting REM via generalized linear mixed modeling using currently available standard procedures in SAS (e.g. PROC GLIMMIX) was very difficult and same problems exist in Stata’s gllamm or R’s lme packages. Thus, this study proposes and assesses the performance of a meta regression approach and makes comparison with methods based on sampling of the full data.

**Data:**

We use both simulated and real data from a national cohort of Veterans with type 2 diabetes (n=890,394) which was created by linking multiple patient and administrative files resulting in a cohort with longitudinal data collected over 5 years.

**Methods and results:**

The outcome of interest was mean annual HbA1c measured over a 5 years period. Using this outcome, we compared parameter estimates from the proposed random effects meta regression (REMR) with estimates based on simple random sampling and VISN (Veterans Integrated Service Networks) based stratified sampling of the full data. Our results indicate that REMR provides parameter estimates that are less likely to be biased with tighter confidence intervals when the VISN level estimates are homogenous.

**Conclusion:**

When the interest is to fit REM in repeated measures data with very large sample size, REMR can be used as a good alternative. It leads to reasonable inference for both Gaussian and non-Gaussian responses if parameter estimates are homogeneous across VISNs.

## Background

Many translational research projects are generating very large data sets (VLDS) which require fitting complex models to answer questions of public health interest. Datasets can be considered “very large” because of large numbers of study subjects or units of analysis and/or large numbers of variables, and both situations present challenges during the analysis phase, especially when observations are clustered at some level (eg. Longitudinal data). An example of VLDS with large number of observations is a two-year group randomized trial designed to assess the impact of a quality improvement intervention on colorectal cancer screening in primary care practices. Electronic medical record data were obtained from a sample of 68,150 patients from 32 primary care practices in 19 US states, followed monthly over a 2-year time period [1]. Similarly, an example of VLDS with large number of variables as well as units of analysis is an functional magnetic resonance imaging study of neural changes underlying speech-perception training [2] in which whole brain images of 40 patients were taken to make functional inference, resulting in hundreds of time series data clustered within thousands of voxels.

Fitting complex models for these types of data sets can be difficult, requiring inordinate amounts of computer time for parameter estimation, requiring memory allocations beyond what are available or containing data structures that prevent model convergence, even within state-of-the-art computational infrastructures of medium size research facilities such as ours. For instance, fitting complicated generalized linear mixed models (GLMMs) for data from the examples above using software such as SAS 9.2.2 (Cary, NC), Stata 11 (College Station, TX) or R (R-2.11.1) may not be possible using desktop computers typically available to researchers within our institutions (64 bit server with 12GB and 667MHz dual ranked DIMMS and 48GB of RAM). Although a few methods for modeling VLDSs exist, current practice mainly involves data reduction processes, which usually result in loss of information.

Recently, we have been working on a longitudinal study of the trajectory of HbA1c control in patients with type2 diabetes treated within the Veterans Administration (VA) healthcare setting, and we have been faced with the problem of fitting GLMMs on over 890,000 patients, clustered in 23 Veterans Integrated Service Networks (VISNs) and followed over 5 years. Fitting mixed effects logistic regression model with over 30 covariates for making individual level inference resulted in an out of memory error using a 64 bit server with 12GB and 667MHz dual ranked DIMMS and 48GB of RAM.

In SAS procedures such as Proc GLIMMIX, fitting mixed effect models with the recommended standard syntax of including subject ID in a Class statement was not possible. This procedure with the standard syntax ran out of memory when we attempted to fit a model with the simplest scenario of including a random intercept. With ad-hoc modifications (see discussion section) to the standard syntax, however, we were able to fit the model despite it took longer time. Similar problems were observed in Stata’s gllamm, and R’s lme4 packages.

With the current focus on personalized medicine, patient/subject level inference is often of key interest in translational research. GLMMs are a very rich class of models that are traditionally used to make such individual-level inference by breaking down the total variation in the observed response into within-subject and between-subject variation. These models are also used to incorporate natural heterogeneity in the estimates due to unmeasured explanatory variables [3-5]. In GLMMs, the joint distribution of the vector of responses is fully specified and the within-subject association among repeated or clustered measures is induced via incorporation of one or more random effects into the model. As a result, interpretation of the regression coefficients for GLMM relies on the induced model for the covariance among the responses. When population level inference is of interest, marginal models (e.g. general linear models) are often used, and within-subject association among repeated responses is incorporated by directly making assumptions about the covariance (e.g. autoregressive, compound symmetry, etc). While such models may not be as difficult to fit with VLDSs, subject-level inference cannot be made using the marginal model framework since the mean response and covariance are modeled separately [3]. Currently, methodology for fitting parametric mixed effect models for VLDSs is underdeveloped.

There are some recent Bayesian methods proposed for fitting parametric random effects models to VLDSs [6-8]. Owen [9] and Huang and Gelman [7] propose a computational strategy, akin to a Bayesian meta regression, based on sampling the data, computing separate posterior distributions based on each sample, and then combining these to get a consensus posterior inference. Their approach reduces the number of parameters as well as sample size for each separate model fit and can lead to efficient inference.

An alternative is a 2-stage “data squashing” method [10]. In this method, the complete data is partitioned into compact sub-regions in the first stage. Then one generates a set of “pseudo-data” and weights within each sub-region so that the weighted moments on the squashed data match the unweighted moments on the original data. This method is less sensitive to outliers than random sampling, but it has the potential to be computationally intensive. To date, its characteristics are only known in simpler fixed effect and descriptive models. Madigan *et al*. [11,12] proposed a data squashing method which first groups subjects based on their contribution to the likelihood and then fits models to the mean of each group. Although this approach may be promising for some models, it is unwieldy under the Dirichlet process prior (DPP) due the complicated structure of the likelihood [12]. In general, the Bayesian approaches which use DPP to automatically cluster individuals into latent classes [13,14] may not be feasible in very large data sets due to limitations in current Markov chain Monte Carlo (MCMC) algorithms [12,15].

Motivated by the scarcity of work in this area and the challenge we faced with the analysis of our VLDS, we propose a random effects meta regression (REMR) approach in which VISN-specific estimates are combined via meta regression. We make comparisons with two other approaches, (1) average estimates from analysis of 1000 data sets obtained via simple random sampling (SRS) of the original data with simulated 95% confidence intervals (CIs), (2) weighted average estimates from analysis of 1000 data sets obtained via VISN-stratified random sampling (StRS) with simulated 95% CIs. Using simulated data, we also assess biases present within each approach, noting whether they provide equivalent inferences as would be obtained from analysis of the full data. The paper is organized as follows: section 2 presents the motivating example; section 3 describes the details of the statistical methods; section 4 presents the results of the analysis; and section 5 discusses the findings.

### Motivating example

A national cohort of Veterans with type 2 diabetes was created by linking patient and administrative files from the Veterans Health Administration (VHA) National Patient Care and Pharmacy Benefits Management (PBM) databases. Veterans were included in the cohort if they had type 2 diabetes defined by two or more International Classification of Diseases, Ninth Revision (ICD-9) codes for diabetes (250, 357.2, 362.0, and 366.41) in the previous 24 months (2000 and 2001) and during 2002 from inpatient stays and/or outpatient visits on separate days (excluding codes from lab tests and other non-clinician visits), and prescriptions for insulin or oral hypoglycemic agents (VA classes HS501 or HS502, respectively) in 2002 [16]. Veterans identified as having type 2 diabetes by ICD-9 codes were excluded from the cohort if they did not have prescriptions for diabetic medications (HS501 or HS502) in 2002. The datasets were linked using patient scrambled Social Security Numbers and resulted in 890, 394 Veterans, who were followed until death, loss to follow-up, or through December 2006. The study was approved by our Institutional Review Board and local VA Research Development committee.

#### Outcome measure

The primary outcome was glycosylated hemoglobin (HbA1c) level. In addition, a binary outcome defined as HbA1c ≥ 8.0% was used.

#### Primary independent variable

For this project, the primary research question was whether HbA1c differed significantly by race/ethnicity, classified as non-Hispanic white (NHW), non-Hispanic black (NHB), Hispanic, and other/unknown/missing.

#### Demographic variables

Age, gender, marital status (i.e., single or married) and percentage service-connectedness (i.e., degree of disability due to illness or injury that was aggravated by or incurred in military service) were available and treated as covariates in the model. Location of residence was defined as urban and rural/highly rural, [17] and hospital region was defined by the five geographic regions of the country based on VHA Veteran’s Integrated Service Networks (VISNs): Northeast (VISNs 1, 2, & 3), Mid-Atlantic (VISNs 4, 5, 6, 9, & 10), South (VISNs 7, 8, 16, & 17), Midwest (VISNs 11, 12, 15, 19, & 23), and West (VISNs 18, 20, 21, & 22) [18].

#### Comorbidity

Variables included substance abuse, anemia, cancer, cerebrovascular disease, congestive heart failure, cardiovascular disease, depression, hypertension, hypothyroidism, liver disease, lung disease, fluid and electrolyte disorders, obesity, psychoses, peripheral vascular disease, and other (AIDS, rheumatoid arthritis, renal failure, peptic ulcer disease and bleeding, weight loss) and were defined based on ICD-9 codes at entry into the cohort. In our final models, we included a categorical summary of count of comorbidities defined as (0=none, 1=one, 2=two 3=three or more), a process which has been shown to be as or more efficient than more complicated algorithms [19].

## Methods

### Overview of the generalized linear mixed model (GLMM)

To model the relationship between HbA1c (Y) and covariates (X), a GLMM approach was used. For the ith subject (i=1,.,N) with n_i_ (j=1,…,n_i_) repeated measurements, we considered the model, E(Y_i_ |X_i_,Z_i_) =g^-1^(X_i_β + Z_i_b_i_), where g is a monotone link function and Y_i_ is Nx1 vector of responses, X_i_ is n_i_xp matrix of covariates, Z_i_ is n_i_xq matrix of covariates (q≤p), β is a px1 vector of fixed effect parameters, b_i_ is a qx1 vector of random effects. We assume that b_i_~N(O,G), where G is a qxq covariance matrix for b_i_. An identity link function results in a linear mixed model for the continuous HbA1c outcome, and a logit link results in logistic mixed effects model for the dichotomous HbA1c outcome. If b_i_ is a vector of random intercept and slope, it results in a 2x2 covariance matrix G which indicate natural heterogeneity among individuals in both their baseline level and changes in the expected outcomes over time. In our models, a person-level random effect was included in all models to account for within-individual correlations. This approach accommodates a wide range of distributional assumptions, multilevel data, measurement of subjects at different time points, modeling individual level effects, missing data, and time varying or invariant covariates [20].

A special case is the linear mixed model given by, Y_i_ |X_i_,Z_i_ =X_i_β + Z_i_b_i_ + e_i_, where e_i_~N(0,R_i_) and independent of b_i_. Assuming, R_i_=σ^2^I_ni_, the conditional distribution of Y_i_|b_i_ is given by the multivariate Gaussian distribution with mean Z_i_b_i_ +X_i_β and variance σ^2^I_ni_. In this model, the response for the i^th^ subject is assumed to differ from the population mean, E(Y_i_)=X_i_β, by a subject specific effect, Z_i_b_i_, and a within-subject measurement error e_i_. The estimates of the parameters in a mixed model are determined as the values that optimize an objective function which is either the likelihood of the parameters given the observed data (ML) or a related objective function called the restricted ML (REML). In practice REML is often preferred. The log-likelihood based on the observed data assuming that the vector of all variance components in G and R_i_ can be denoted by α can be written as,

(1) $$\begin{array}{l} l\left(\beta ,\alpha \right)=\sum_{i=1}^{N}-\frac{n_{i}}{2}\log\left(2\pi \right)-\frac{1}{2}\sum_{i=1}^{N}\log|\Sigma_{i}\left(\alpha \right)|\\ \;-\frac{1}{2}\sum_{i=1}^{N}{\left(y_{i}-X_{i}\beta \right)}^{\prime }\Sigma_{i}{\left(\alpha \right)}^{-1}\left(y_{i}-X_{i}\beta \right) \end{array}$$

where *Σ*_*i*_(*α*) = *Z*_*i*_*G*(*α*)*Z*_*i*_^′^ + *R*_*i*_(*α*) and parameter estimates are obtained via Newton-Raphson.

### Weighted Generalized Linear Mixed Effects Model (WGLMM)

The WGLMM is a model well-suited for analysis of survey sampled data. We use it to analyze our type-2 diabetes cohort data in the context of finitely sampled data (e.g. VISN-stratified randomly sampled data).

In sample surveys, units are sometimes drawn with unequal selection probabilities, and if the design probabilities are informative (i.e. they are related to the response) [21,22] the model holding for the sample will be different from the model holding for the finite population. Consequently, the usual estimators will be biased for the finite population quantity [23,24]. A common design based solution is to use weighted estimators where the contribution of unit *i* is weighted by the inverse probability of selection into the sample [25,26]. An alternative is a model based approach where the sampling units enter into the model as random effect terms. In the former, a pseudolikelihood approach for accommodating inverse probability weights is implemented by using adaptive quadrature, and a sandwich estimator is used to obtain standard errors that account for complex sampling, since model-based standard error estimates may not be valid. These types of models can be fitted using SAS procedures such as Proc GLIMMIX by including weights via the WEIGHT statement. Similarly, xtmixed and gllamm can be used in Stata and the lme4 package in R. In the Stata program gllamm, a full pseudo-maximum-likelihood estimation which allows for specification of probability weights, is implemented via adaptive quadrature [27]. The weights enter the log-pseudolikelihood as if they were frequency weights, representing the number of times that each unit should be replicated. Adaptive quadrature [27,28] provides good approximations to the integrals in the pseudolikelihood. However, these are not easy to implement in practice since the log-pseudolikelihood cannot simply use one set of weights based on the overall inclusion probabilities but must use separate weights at for the fixed and random effects.

The generalizations of Equation (1) above to the special case of weighted linear mixed model (WLMM) can be described by the change in the conditional distribution (Y_i_|b_i_)~N( Z_i_b_i_ +X_i_β ;R_i_(α)W_i_^-1^) and *Σ*_*i*_(*α*) = *Z*_*i*_^′^*W*_*i*_^− 1^*G*(*α*)*Z*_*i*_ + *R*_*i*_(*α*)*W*_*i*_^− 1^ where the weights matrix, W, is constant. In our implementation of WLMM, we used a residual subject specific pseudolikelihood (RSPL) to estimate parameters and simulated 95% CIs which are calculated based on 1000 simulations.

### Meta regression approach

Another approach to deal with fitting parametric random effects models to VLDS is to do aggregated analysis after estimating the parameters at some level of administrative or sampling based subsets of the data. This can lead to substantial gain in the time required to fit these models and can be adapted to parallel processing, leading to further computational time savings. In the case of likelihood inference, this idea leads to a pseudolikelihood [29-31], where weights are incorporated as if they were frequency weights. The resulting estimator is design consistent and hence model consistent under suitable regularity conditions such as those discussed by Isaki and Fuller [32]. However, this consistency typically comes at a price of reduced efficiency [33].

Since VHA research data are provided at VISN level, models for each VISN can be fitted, and a mechanism to combine these parameter estimates is suggested. After models relating HbA1c and covariates are fitted for each VISN, the next step is to use pooling methods to obtain national estimates. This can be done using fixed effects [34,35] and random effects meta regression [36-38]. The fixed effects approach [39] is based on weighted regression with the weight being the number of patients in each VISN as fraction of the total population. Covariates can either be VISN level factors or aggregates of individual level covariates. The second approach is random effects meta-regression [35]. Generally the regression coefficients are regressed on an intercept and VISN-level covariates. A random intercept is included in the regression to take into account the between-VISN variation. This leads to the usual DerSimonian and Laird [40] random effects estimate of the pooled regression coefficient.

### Fixed effects meta regression (FEMR)

Let ψ_i_ be an effect of interest to estimate for VISN i. In our study these are the regression coefficients associated with covariates such as race/ethnicity in the GLMM model. Let ϕ_i_ be the corresponding sample estimate. The fixed effects meta regression can be given by ϕ_i_ =τ +ε_i_, where τ is the pooled mean or FEMR estimate and ε_i_~N (0, σ_i_^2^) is the random error. This can be adjusted for covariates via weighted regression as:

(2) $$\phi_{i}=\tau +\gamma_{1}z_{i}+\gamma_{2}x_{i}+\varepsilon_{\text{i}},\text{where}\varepsilon_{i}∼N\left(0,{\sigma_{i}}^{2}\right)$$

### Random effects meta regression (REMR)

In REMR, a standard one-step DerSimonian and Laird [40] random effects pooled estimate of the regression coefficient is obtained via, ϕ_i_ =τ +ν_i_ +ε_i_ , where ε_i_~N (0, σ_i_^2^) and ν_i_~N (0, σ_v_^2^) with v_i_ (VISN level random effect) and ε_i_ (random error) are uncorrelated. In the covariate adjusted version, the model is fitted by regressing the effect estimate on an intercept and VISN-level covariates:

(3) $$\begin{array}{l} \phi_{i}=\tau +\gamma_{1}z_{i}+\gamma_{2}x_{i}+\nu_{i}+\varepsilon_{i},\\ \;\text{where}\varepsilon_{i}∼N\left(0,{\sigma_{i}}^{2}\right)\text{and}\;\nu_{i}∼\text{N}\left(0,{\sigma_{v}}^{2}\right) \end{array}$$

where τ is the pooled mean or REMR estimate. The adjustment covariates can be VISN level covariates (z) or aggregates of individual level covariates (x) to account for additional causes of heterogeneity [41]. For both fixed effects [34] and random effects meta regression [37,38], we used restricted maximum likelihood via Proc GLIMMIX and Proc PLM (SAS 9.2.2) to obtain pooled estimates.

### Summary of modelling strategies used

In this paper, we study two broad strategies for longitudinal analyses of VLDS: random effects meta regression (REMR) and estimation based on sampling of the full data (SRS and StRS). Within each strategy we model the continuous outcome of HbA1c using a linear mixed model and the binary outcome of HbA1c (<8% vs. ≥ 8%) using mixed effects logistic regression. The primary independent variable is race/ethnicity, and a number of subject-level covariates are included.

### Test of homogeneity

The main goal of REMR and FEMR is to obtain a single global or pooled effect summarized across VISNs. But, obtaining pooled estimates assumes homogeneity of VISN level effects. According to [42], the main sources of heterogeneity are clinical incomparability or design incomparability. Clinical incomparability can be caused by differences in the VISN level populations, and design incomparability can be caused due to differences, for example, in missing data and measurement error. These are issues, however, that mainly arise in pooling of effects from different studies. In our data, these issues may not arise at all or will have very limited impact on generalizability of results. If heterogeneity is found or suspected to exist, the common approaches used in meta-analysis are (1) to stratify the studies into homogeneous subgroups and then fit a separate fixed effects estimate [43], (2) construct a random effects estimate [40] across all VISNs (a random effects approach incorporates both within-study and between-study variability: if heterogeneity exists among VISNs, a summary measure across those VISNs may not be provided), or (3) fit a meta-regression model that explains the heterogeneity in terms of VISN-level covariates. We implemented an approach of removing outliers, as suggested by Draper and Smith [44] in conjunction with approach 2 above.

### Model selection

Although the purpose of this project was not to “select” an optimal model, model fit assessment was facilitated using maximum likelihood (or pseudo-likelihood) information criteria, factors typically used in model selection. Two common approaches in the literature include Akaike information criterion (AIC) [45] and Bayesian information criterion (BIC) [46]. Across competing models the lowest value on each criterion indicates the best fitting model. These statistics account for both model fit (deviance) and model complexity by penalizing models with a larger number of parameters. For GLMM, pseudo-AIC and pseudo-BIC, which are adjusted for fixed effects and covariance parameters, are used. However these pseudo information criteria are not useful for comparing models that differ in their pseudo data [3]. Thus in our example, they are used to compare AIC/BIC values of models estimated using the sampled data and to the full data models. AIC/BIC values from REMR are not comparable with those from the full data.

### Bootstrap simulation study design

Simulation studies based on 1000 repeated re-samplings of (sample size: 1%, 5%, 10% and 25%) the full data are used to asses and compare the methods discussed above. This is implemented via a non-parametric bootstrapping approach [47] with a repeated sample of the observed data with replacement with the study subject as the sampling unit to form 1000 simulated datasets of each sample size. Traditionally, Monte-Carlo simulation studies based on data generated from statistical models have been used for this kind of comparative study. Resampling has the advantage that the data in resampled datasets are based on observations from real datasets and thus reflect the appropriate level of diversity and variability found in realistic populations [47,48]. Our datasets are large enough to permit numerous samples of reasonable size to arrive at stable conclusions within the resampled data [47]. Performance of methods in terms of bias and efficiency will be benchmarked against results from the original full data. The full data estimates are used as true values to judge bias in estimates from the sampling and meta regression approaches.

### Hardware

All analyses for this investigation were run on a Dell PowerEdge 2900 III server with two dual core Intel Xeon X5260 processors with 6 megabyte cache, with a clock speed of 3.33 gigahertz, and a front-side bus of 1333 megahertz. The server has been configured with 12 four gigabyte (GB), 667MHz dual ranked dual in-line memory modules for a total of 48GB of RAM. Data are stored on six one terabyte (TB) 7200 revolution per minute near-line serial attached small computer system interface, 3GB per second 3 ½ inch HotPlug hard drives forming a 3TB redundant array of independent disk level 5 storage system. This server runs a 64-bit version of Windows 2003 R2 Enterprise X64 Edition Service Pack 2 operating system.

### Software

Datasets were organized for this study using SAS version 9.2.2 (Cary, NC) and SAS transport data sets created. Data were read into a 64-bit version of R for Windows 2.11.1 (R Development Core Team 2010) using the “Hmisc” [49] and “foreign” [50] packages. Plots were done using R, and regression analysis was accomplished using the lme4 package [51].

## Results

The full cohort consisted of 890,394 Veterans with diabetes followed from 2002 through 2006. The cohort is characterized based on demographics, HbA1c and comorbidities in Table 1. The full cohort is 62% NHW, 12% NHB, 14% Hispanic, and 12% other race/ethnicity. Characteristics of the 1%, 5%, 10% and 25% random samples estimates as well as REMR reflect that of the full cohort, with prevalence estimates across the four sampling scenarios (1%, 5%, 10% and 25%) and the full cohort differing by no more than 1%. The mean age in the full cohort is 66.2 years and ranges from 66.2 years in the 10% and 25% SRS to 66.5 years in the 1% SRS.

**Table 1:** Characteristics of study population for the full (n=890,394) and sampled cohorts

**Analysis variable**	**Full cohort (n=890,394)**		**25% (n=225,000)**		**10% (n=90,000)**		**5% (n=45,000)**		**1% (n=9,000)**		**REMR (n=890,394)**	
Non-Hispanic White: % (n)	62	(547,645)	61	(138,470)	62	(55,489)	62	(27,853)	62	(5,529)	62	(547,645)
Non-Hispanic Black: % (n)	12	(107,935)	12	(27,317)	12	(10,941)	12	(5,406)	12	(1,097)	12	(107,935)
Hispanic: % (n)	14	(123,558)	14	(31,062)	14	(12,481)	14	(6,148)	14	(1,285)	13	(123,558)
Other: % (n)	12	(111,256)	13	(28,151)	12	(11,089)	12	(5,593)	12	(1,089)	13	(111,256)
Male: % (n)	98	(869,508)	98	(219,708)	98	(87,921)	98	(43,947)	98	(8,794)	98	(869,508)
Married: % (n)	65	(574,307)	64	(145,060)	65	(58,222)	64	(29,002)	65	(5,853)	64	(574,307)
Disability (mean % & sd)	12	(0.03)	12	(0.06)	12	(0.09)	12	(0.13)	13	(0.30)	12	(0.63)
Northeast	12	(103,056)	12	(25,994)	11	(10,274)	12	(5,272)	12	(1,074)	-	(103,056)
Mid-Atlantic	23	(201,058)	22	(50,579)	23	(20,328)	23	(10,230)	23	(2,000)	-	(201,058)
Midwest	21	(184,348)	21	(46,940)	21	(18,658)	21	(9,368)	20	(1,827)	-	(184,348)
South	30	(265,450)	30	(66,988)	30	(26,759)	29	(13,189)	30	(2,707)	-	(265,450)
West	15	(136,482)	15	(34,499)	16	(13,981)	15	(6,941)	16	(1,392)	-	(136,482)
Urban Residence	62	(548,786)	61	(138,339)	61	(55,324)	62	(27,701)	61	(5,513)	61	(548,786)
Rural Residence	38	(341,608)	39	(85,612)	39	(34,676)	38	(17,299)	39	(3,487)	39	(341,608)
Mean HbA1c (mean % & sd)	7.4	(0.002)	7.4	(0.003)	7.4	(0.005)	7.4	(0.007)	7.4	(0.016)	7.5	(0.030)
Mean HbA1c<8%: % (n)	73	(703,596)	73	(177,751)	73	(71,195)	73	(34,498)	71	(7,112)	70	(703,596)
No Comorbidities	57	(507,320)	57	(128,326)	57	(51,178)	57	(25,506)	57	(5,143)	57	(507,320)
1 Comorbidity	28	(248,898)	28	(62,961)	28	(25,309)	28	(12,655)	27	(2,456)	28	(248,898)
2 Comorbidities	11	(95,542)	11	(23,998)	11	(9,706)	11	(4,898)	11	(1,022)	11	(95,542)
3+ Comorbidities	4	(38,634)	4	(9,715)	4	(3,807)	4	(1,941)	4	(379)	4	(38,634)

Not applicable due to sampling by VISN or aggregation by VISN.

Linear mixed models were used to model continuous HbA1c levels in both the full cohort and each of the SRS and StRSs (using the weighted approach described in section 3.2). Parameter estimates associated with betas representing the different race/ethnic and comorbidity groupings, standard errors of the betas, and 95% confidence intervals are reported in Tables 2. For all variables examined with the exception of comorbidity, the direction of the association between each independent variable and HbA1c was consistent in both SRS and StRS with the full cohort. In fact, the beta estimates for all the 1% to 5% SRSs or StRS scenarios were within the 95% confidence limits of the full cohort estimates (1% estimates are removed from Tables to save space). For example, the beta estimate for NHB indicated that HbA1c levels were 0.46 units higher in NHB than NHW in the full cohort, while estimates in the SRSs ranged from 0.39 in the 1% SRS to 0.46 in the 25% SRS. The estimates for NHB in the StRS also ranged from 0.44 in the 1% sample to 0.47 in the 25% sample. However, for the number of comorbidities variable, while the direction of the beta estimate remained similar between the full cohort and the SRS or StRS, the magnitude and 95% CI for the 1% sample was higher, and the association was not statistically significant (Table 2). For example, the *β* estimate (95% confidence intervals) for three or more comorbidities was 0.11 (0.11, 0.13) for the full cohort and it ranged between 0.04 (-0.05, 0.16) in the 1% SRS to 0.10 (0.08, 0.13) in the 25% SRS and 0.18 (0.08, 0.30) in the 1% StRS to 0.11 (0.1, 0.15) in the 25% StRS. The smaller standard errors and tighter confidence intervals observed in the full cohort are a direct result of the larger sample size.

**Table 2:** Parameter estimates, 95% confidence intervals, standard errors for intercept, race and comorbidity in linear mixed model (LMM^*^) of HbA1c using sampling and random effects Meta-regression, in for Veterans with Type 2 Diabetes (2002-2006)

**Simple random sample (SRS)**								
**Parameter**	**Sample (%)**	**Intercept**	**Non-hispanic black**	**Hispanic**	**Other**	**1 Comorbidity**	**2 Comorbidities**	**3+ Comorbidities**
β (95% CI)	100	7.54 (7.52, 7.55)	0.46 (0.45, 0.46)	0.29 (0.28, 0.30)	0.25 (0.23, 0.25)	0.01 (0.01, 0.02)	0.04 (0.04, 0.05)	0.11 (0.11, 0.13)
	25	7.59 (7.55, 7.61)	0.46 (0.44, 0.47)	0.31 (0.28, 0.32)	0.24 (0.22, 0.25)	0.01 (0.00, 0.02)	0.02 (0.01, 0.04)	0.10 (0.08, 0.13)
	10	7.54 (7.48, 7.58)	0.47 (0.44, 0.48)	0.30 (0.26, 0.32)	0.26 (0.23, 0.27)	0.03 (0.02, 0.05)	0.08 (0.07, 0.12)	0.08 (0.05, 0.13)
	5	7.54 (7.48, 7.62)	0.44 (0.41, 0.47)	0.28 (0.23, 0.32)	0.27 (0.24, 0.30)	0.03 (0.01, 0.06)	0.05 (0.02, 0.09)	0.13 (0.08, 0.18)
SE	100	0.0115	0.005	0.007	0.005	0.0037	0.0054	0.0079
	25	0.0115	0.005	0.007	0.005	0.0037	0.0054	0.0080
	10	0.0115	0.005	0.007	0.005	0.0037	0.0054	0.0080
	5	0.0116	0.005	0.0069	0.005	0.0037	0.0053	0.0079
**Stratified random sampling (StRS)**								
Parameter	Sample (%)	Intercept	Non-Hispanic Black	Hispanic	Other	1 Comorbidity	2 Comorbidities	3+ Comorbidities
β (95% CI)	25	7.61 (7.57, 7.63)	0.47 (0.45, 0.48)	0.28 (0.26, 0.29)	0.26 (0.24, 0.27)	0.01 (0, 0.02)	0.03 (0.02, 0.05)	0.11 (0.11, 0.15)
	10	7.58 (7.53, 7.63)	0.46 (0.43, 0.48)	0.28 (0.25, 0.30)	0.26 (0.23, 0.28)	0.0 (-0.01, 0.02)	0.05 (0.03, 0.08)	0.16 (0.13, 0.2)
	5	7.61 (7.54, 7.68)	0.38 (0.35, 0.41)	0.30 (0.26, 0.35)	0.25 (0.21, 0.28)	0.02 (0.0, 0.05)	0.05 (0.02, 0.09)	0.09 (0.05, 0.15)
SE	25	0.0111	0.0049	0.0068	0.005	0.0037	0.0054	0.0079
	10	0.0111	0.0049	0.0068	0.0049	0.0037	0.0054	0.0078
	5	0.0111	0.0050	0.0069	0.005	0.0037	0.0053	0.0079
**Random effects meta-regression without VISN 13 & 14 (REMR)**								
Parameter^**^	Sample (%)	Intercept	Non-Hispanic Black	Hispanic	Other	1 Comorbidity	2 Comorbidities	3+ Comorbidities
β(95% CI)	100	7.58 (7.54, 7.62)	0.45 (0.41, 0.49)	0.08, (0.04, 0.12)	0.23 (0.19, 0.27)	0.01 (-0.04, 0.05)	0.03 (-0.01, 0.07)	0.09 (0.05, 0.13)

*-Independent variables used in fitting the linear mixed model were: linear time; race (non-Hispanic white reference, indicator variables); sex (female reference); marital status (single reference), service disability percentage, residence status (urban/rural, rural reference), VISN region (Northeast, Mid-Atlantic, South, Midwest, and West), and number of comorbidities (1, 2, or 3+; none reference).

** Veteran Integrated Service Networks (VISNs) 13 and 14 are excluded in all these models.

The REMR estimates on the other hand are very close to the full sample estimates. For example, the beta estimate for NHB indicated that HbA1c levels were 0.45 (0.41, 0.49) higher in NHB than NHW in REMR which is comparable to 0.46 (0.45, 0.46) in the full cohort. Similarly, for three comorbidities the full cohort results were 0.11 (0.11, 0.13) while REMR resulted in 0.09 (0.05, 0.13). In all these models, the intercept was very well approximated even in the 1% sampled data. It should be noted that, REMR can be highly affected by outliers in the estimates that are aggregated to get the final estimates. In our case, VISN 13 and 14 exhibited extreme values and hence were removed to maintain the homogeneity assumption required by REMR in order to get unbiased estimates. In Table 2, the REMR estimates as well as their 95% CI are very similar to the full sample estimates reported in the first row of the table except that the full data 95% CI estimates are much tighter as expected. REMR models that included VISN 13 and 14 are summarized in Additional file 1: Appendix Tables 1 and 2. These appendix tables show that REMR can lead to biased estimates when the assumption of homogeneity of VISN level estimates is violated.

Table 3 compares results from GLMM for binary HbA1c for the three different approaches considered. While it was not possible using standard GLMMIX syntax to obtain estimates for the full sample or for samples of size 10% or more (due to well known computational convergence and memory limitation errors), we were able to obtain estimates for the full sample using tricks such as sorting by ID and time and removing them from the class statement. However, it was not possible to obtain estimates using R’s lme4 package. Compared to NHW, the REMR estimate (and 95% CIs) for the log odds of HbA1c>8% was 0.58(0.52, 0.64) for NHB, 0.11(0.05,0.17) for Hispanic and 0.32(0.26,0.38) for Other race/ethnic groups. These mimic the full sample estimates reported in the first row of Table 3 showing that REMR estimates (when homogeneity assumption is met) lead to unbiased estimates. On the other hand, the estimates from both SRS and StRS were very biased with bias increasing inversely with sample size. The corresponding estimates (and 95% CIs) of the log-odds ratio in the 5% SRS were 1.69(1.40,1.99), 1.10(0.70,1.50) and 1.05(0.75,1.35) for NHB, Hispanic, and Other respectively. The results from StRS were similar to SRS. On the other hand, while the REMR log-odds ratio estimate for having three or more comorbidities was 0.25(0.19,0.31), the corresponding estimates from 5% SRS and StRS were 0.33(-0.24,0.90) and 0.80(0.22,1.39) respectively. The estimates with VISNs 13 and 14 included are in Additional file 1: Appendix Tables 1 and 2, indicating even worse performance by these sampling approaches. 

**Table 3:** Parameter estimates (95% CI), standard errors for intercept, race and comorbidity in general linear mixed model (GLMM^†^) for binary HbA1c using sampling and random effects meta-regression, in for veterans with type 2 diabetes (2002-2006)

**Simple random sample (SRS)**								
**Parameter**	**Sample (%)**	**Intercept**	**Non-hispanic black**	**Hispanic**	**Other**	**1 Comorbidity**	**2 Comorbidities**	**3+ Comorbidities**
β (95% CI)	100	-0.94 (-0.98, -0.91)	0.62 (-0.02, -0.01)	0.45 (0.43, 0.48)	0.36 (0.35, 0.38)	0.07 (0.06, 0.08)	0.15 (0.13, 0.17)	0.27 (0.24, 0.29)
	25	-1.93 (-2.17, -1.69)	1.27 (1.17, 1.37)	1.07 (0.93, 1.20)	0.87 (0.77, 0.97)	0.12 (0.05, 0.21)	0.19 (0.06, 0.31)	0.39 (0.19, 0.58)
	10	-2.48 (-2.93, -2.04)	1.39 (1.20, 1.58)	1.27 (1.01, 1.53)	0.97 (0.78, 1.15)	0.16 (0.01, 0.31)	0.36 (0.14, 0.59)	0.44 (0.07, 0.80)
	5	-2.16 (-2.87, -1.45)	1.69 (1.40, 1.99)	1.10 (0.70, 1.50)	1.05 (0.75, 1.35)	0.39 (0.16, 0.62)	0.34 (0.09, 0.80)	0.33 (-0.24, 0.90)
SE	100	0.0182	0.0076	0.0105	0.0078	0.0053	0.0085	0.0125
	25	0.1221	0.0514	0.0691	0.0512	0.0402	0.0621	0.3896
	10	0.2269	0.0965	0.1308	0.0959	0.0748	0.1163	0.1858
	5	0.3608	0.1505	0.2038	0.1520	0.1175	0.1821	0.2905
**Stratified random sampling (StRS)**								
Parameter	Sample (%)	Intercept	Non-Hispanic Black	Hispanic	Other	1 Comorbidity	2 Comorbidities	3+ Comorbidities
β (95% CI)	25	-1.83 (-2.07, -1.59)	1.25 (-0.01, 0.01)	0.90 (0.76, 1.03)	0.88 (0.78, 0.98)	0.10 (0.02, 0.18)	0.30 (0.17, 0.42)	0.75 (0.56, 0.94)
	10	-2.34 (-2.78, -1.89)	1.47 (1.29, 1.66)	1.03 (0.78, 1.29)	1.00 (0.81, 1.19)	0.07 (-0.08, 0.21)	0.24 (0.02, 0.47)	0.69 (0.34, 1.05)
	5	-2.65 (-3.35, -1.95)	1.44 (1.14, 1.74)	1.74 (1.34, 2.15)	1.10 (0.81, 1.40)	0.20 (-0.03, 0.43)	0.16 (-0.19, 0.52)	0.80 (0.22, 1.39)
SE	25	0.1209	0.0514	0.0690	0.0512	0.0401	0.0620	0.0984
	10	0.2272	0.0955	0.1282	0.0949	0.0745	0.1154	0.1813
	5	0.3561	0.1531	0.2060	0.1505	0.1175	0.1817	0.2984
**Random effects meta-regression without VISN 13 & 14 (REMR)**								
Parameter^**^	Sample (%)	Intercept	Non-Hispanic Black	Hispanic	Other	1 Comorbidity	2 Comorbidities	3+ Comorbidities
β (95% CI)	100	-0.93 (-0.99, -0.87)	0.58 (0.52, 0.64)	0.11 (0.05, 0.17)	0.32 (0.26, 0.38)	0.07 (0.01, 0.13)	0.14 (0.08, 0.20)	0.25 (0.19, 0.31)

†-Independent variables used in fitting the general linear mixed model using a binomial distribution with a logit link function were: linear time; race (non-Hispanic white reference, indicator variables); sex (female reference); marital status (single reference), service disability percentage, residence status (urban/rural, rural reference), and number of comorbidities (1, 2, or 3+; none reference).

** Veteran Integrated Service Networks (VISNs) 13 and 14 are excluded in all these models.

**Figure 1:**
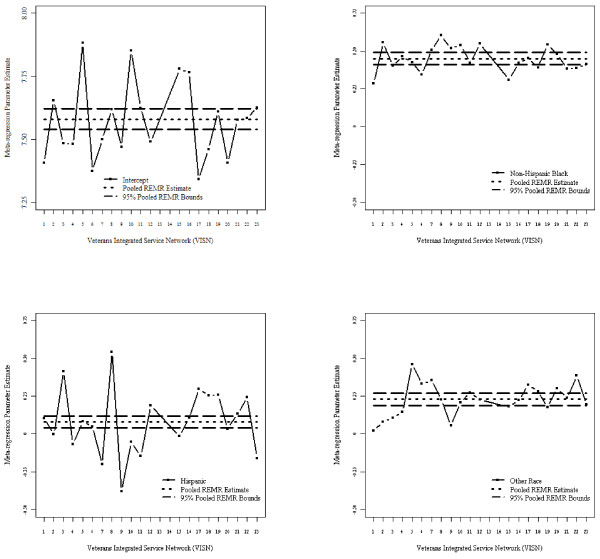
**LMM parameter estimates and pooled 95% confidence bounds for random effects meta-regression (intercept, race) without veteran integrated service networks (VISNs) 13 and 14.** *- Independent variables used in fitting model were: linear time; race (non-Hispanic white reference, indicator variables); sex (female reference); service disability percentage, marital status (single reference), residence status (urban/rural, rural reference), VISN region (Northeast, Mid-Atlantic, South, Midwest, and West, South reference); and number of comorbidities (1, 2, or 3+; none reference).

**Figure 2:**
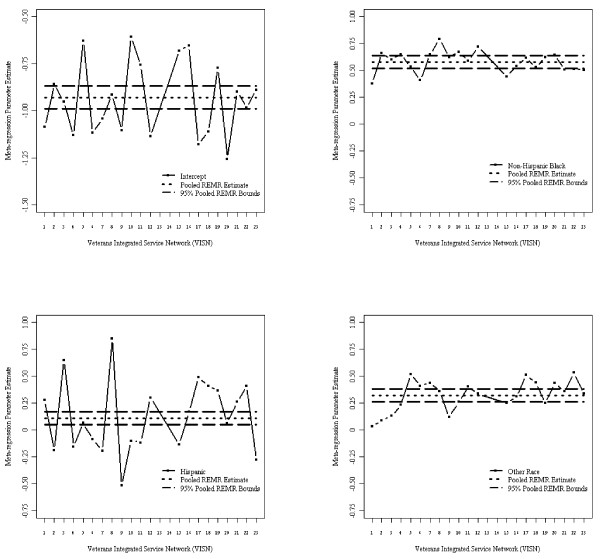
**GLMM parameter estimates and pooled 95% confidence bounds for random effects meta-regression (intercept, race) without veteran integrated service networks (VISNs) 13 and 14.** *- Independent variables used in fitting model were: linear time; race (non-Hispanic white reference, indicator variables); sex (female reference); service disability percentage, marital status (single reference), residence status (urban/rural, rural reference), VISN region (Northeast, Mid-Atlantic, South, Midwest, and West, South reference); and number of comorbidities (1, 2, or 3+; none reference).

**Figure 3:**
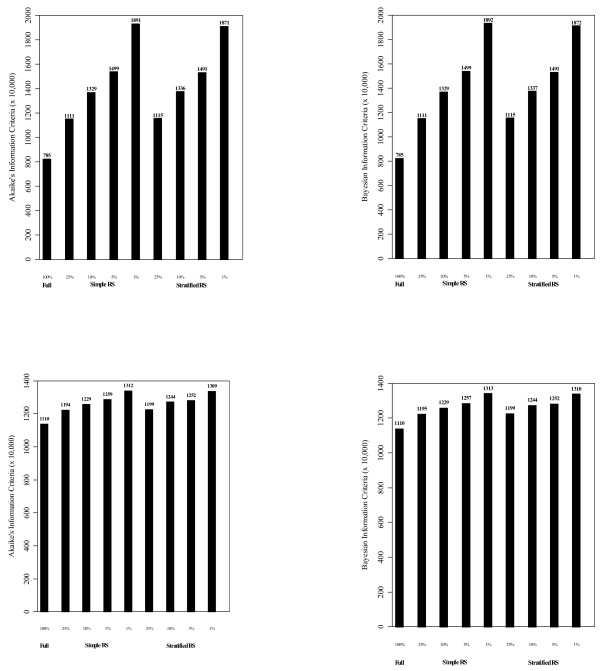
**Akaike’s information criterion (AIC) and Bayesian Information Criterion (BIC) for LMM (top two) and GLMM (bottom two).** *- Independent variables used in fitting the model were: linear time; race (non-Hispanic white reference, indicator variables); sex (female reference); service disability percentage, marital status (single reference), residence status (urban/rural, rural reference), VISN region (Northeast, Mid-Atlantic, South, Midwest, and West, South reference); and number of comorbidities (1, 2, or 3+; none reference).

Figure 1 depicts the beta estimates for race by VISN and the aggregated REMR estimate and their corresponding 95% CI in the association study of HbA1c and race after adjusting for covariates. Figure 2 shows corresponding estimates for binary HbA1c>8% using GLMM. Both figures indicate homogeneity in the estimates across VISNs except in VISNs 13 and 14 (which were removed to meet the homogeneity assumption required by REMR since both VISNs exhibited extreme estimated values). The outlier estimates from VISNs 13 and 14 are clearly depicted in Additional file 1: Appendix Figures S1 and S2. Figure 3 shows the goodness of fit statistics for making comparison among the different models. As expected the AIC and BIC values decreased as sample size increases. Also, AIC/BIC estimates for comparable models in StRS were smaller than those from SRS indicating better fit. While not comparable with the others, the weighted AIC/BIC values for REMR were the smallest indicating better fit.

Additional results corresponding to the analysis of the original full data such as the distribution of subjects in each VISN (Additional file 1: Appendix Table S3), goodness of fit statistics (Additional file 1: Appendix Table S4), how long each model took to fit (Additional file 1: Appendix Table S5) and full covariate models (Additional file 1: Appendix Tables S6 and S7) are in the appendix. Additional file 1: Appendix Table S5 shows that fitting LMM in R’s lme4 package required about 4 times longer time than SAS’s Proc MIXED. Also, while it was not possible to fit GLMM in R, fitting GLMM in Proc GLIMMIX took longer time than obtaining pooled estimates of parameters via REMR.

## Discussion and conclusion

Models with random effects are useful for patient level inference just as marginal models are useful for population level inference. However, for very large data sets, it can be difficult to fit models with random effects using commonly available statistical software such as SAS. There are very few papers on this topic and the most recent work involves a 2-stage Bayesian algorithm [12]. While their method has advantages, there are problems when one needs to adjust for multiple covariates, and it is not clear whether their approach will work in VLDS settings as large as ours.

This study assesses and compares REMR to two sampling based approaches using bootstrap simulation studies. Our results indicate that REMR provides parameter estimates that are less likely to be biased with smaller standard errors when the VISN level estimates are homogenous. The sampling approaches also provide parameter estimates that were equivalent to the full data estimates except when the outcome variable was binary. Thus, when the interest is to fit random effect models in repeated measures data with very large sample size, REMR may be used as a good alternative.

Some ad-hoc approaches can also be considered to ameliorate the challenges with the double optimization required when fitting GLMM to VLDS. For example, SAS Proc HPMIXED is developed to fit LMM to VLDS and provides computational advantages over Proc Mixed in certain situations. Also, sorting the data by variables that need to be in the CLASS statement of Proc MIXED or GLIMMIX, sorting by random effect subject identifiers, may also alleviate the computational burden. However, all of these methods can often not overcome the computational challenges with very large data sets, like those mentioned in the introduction, which makes REMR attractive.

One of the key problems with REMR is handling situations involving heterogeneous parameter estimates. For example, Additional file 1: Appendix Figures S4 and S5 show the estimates for VISNs 13 and 14 which are clearly outliers in the opposite direction. One approach is to remove these outliers, as we did, and obtain unbiased and efficient estimates. The estimates after removing the VISNs with outliers are in Figures 1 and 2. Another approach, illustrated in an extensive simulation study by Morton *et al*. [41,41] is to incorporate important covariates at either the study or person level. However, despite the importance of including covariates, a model that includes a covariate that is an aggregate of a person-level characteristic rather than a study characteristic may also produce biased results. The trade-off between the biases of incorporating an aggregated covariate versus excluding it requires further exploration. While Bayesian random effects meta regression [10,50,51] may be is an alternative, it is not clear how these methods will work for VLDSs and is a topic of future work.

Our work demonstrates a variety of approaches that may be used in analyses of VLDSs, especially when observations are clustered such as in a longitudinal setting. Our simulation results show that SRS and StRS approaches appear to lead to reasonable parameter estimates with Gaussian responses but may be biased when responses are non-Gaussian (eg. Binary). REMR may be an optimal strategy for both Gaussian and non-Gaussian responses, especially when parameter estimates are homogeneous across clusters.

## Abbreviations

CI: Confidence interval; FEMR: Fixed effects meta regression; GLMM: Generalized linear mixed model; LMM: Linear mixed model; NHB: Non-hispanic black; NHW: Non-hispanic white; REM: Random effect model; REMR: Random effects meta regression; SRS: Simple random sample; StRS: Stratified random sample; VISN: Veteran’s integrated service network; VLDS: Very large data sets; VHA: Veteran’s health administration; WGLMM: Weighted GLMM; WLMM: Weighted LMM.

## Competing interests

None of the authors have any financial disclosure or conflict of interest to report.

## Authors' contributions

Study concept and design: MG, Acquisition of data: PM and LE. Analysis and interpretation of data: MG, LE, PM, KH, GG, PN, Drafting of the manuscript: MG, KH, PM, GG, PN, and LE. Critical revision of the manuscript for important intellectual content: MG, LE, PM, PN, and KH. Study supervision: MG and LE. All authors read and approved the final manuscript.

## Pre-publication history

The pre-publication history for this paper can be accessed here:

http://www.biomedcentral.com/1471-2288/12/163/prepub

## Supplementary Material

Additional file 1 **Additional tables and figures that show results for the full model that includes all the covariates under several scenarios are in the appendix.** Another set of tables that include the 1% scenario and REMR results that include VISNs 13 and 14 are in the Appendix. SAS Macro for the procedures we implemented to analyze SRS, StRS and REMR are also available in our website. Click here for file
