# Supplementary material for: Fitting parametric random effects models in very large data sets with application to VHA national data
Source: BMC Med Res Methodol. 2012 Oct 24;12:163. doi: 10.1186/1471-2288-12-163 (PMC3542162; doi:10.1186/1471-2288-12-163)
Supplement: Additional file 1 — Additional tables and figures that show results for the full model that includes all the covariates under several scenarios are in the appendix. Another set of tables that include the 1% scenario and REMR results that include VISNs 13 and 14 are in the Appendix. SAS Macro for the procedures we implemented to analyze SRS, StRS and REMR are also available in our website. [file 1471-2288-12-163-S1.pdf]

Appendix Table 1. Parameter Estimates, 95% Confidence Intervals, Standard Errors for Continuous HbA1c using Simple Random Sampling, Stratified Random Sampling, and Random Effects Meta-regression for Intercept and Race from the Linear Mixed Model (LMM*) for Veterans with Type 2 Diabetes (2002-2006) with VISNs 13 and 14

Simple Random Sample (SRS)								
Parameter	Sample (%)	Intercept	Non-Hispanic Black	Hispanic	Other	1 Comorbidity	2 Comorbidities	3+ Comordbidities
β (95% CI)	100	7.54 (7.52, 7.55)	0.46 (0.45, 0.46)	0.29 (0.28, 0.30)	0.25 (0.23, 0.25)	0.01 (0.01, 0.02)	0.04 (0.04, 0.05)	0.11 (0.11, 0.13)
	25	7.59 (7.55, 7.61)	0.46 (0.44, 0.47)	0.31 (0.28, 0.32)	0.24 (0.22, 0.25)	0.01 (0.00, 0.02)	0.02 (0.01, 0.04)	0.10 (0.08, 0.13)
	10	7.54 (7.48, 7.58)	0.47 (0.44, 0.48)	0.30 (0.26, 0.32)	0.26 (0.23, 0.27)	0.03 (0.02, 0.05)	0.08 (0.07, 0.12)	0.08 (0.05, 0.13)
	5	7.54 (7.48, 7.62)	0.44 (0.41, 0.47)	0.28 (0.23, 0.32)	0.27 (0.24, 0.30)	0.03 (0.01, 0.06)	0.05 (0.02, 0.09)	0.13 (0.08, 0.18)
	1	7.60 (7.42, 7.77)	0.39 (0.32, 0.45)	0.30 (0.22, 0.41)	0.21 (0.14, 0.28)	0.05 (0.01, 0.12)	0.04 (-0.02, 0.13)	0.04 (-0.05, 0.16)
Stratified Random Sampling (StRS)								
Parameter	Sample (%)	Intercept	Non-Hispanic Black	Hispanic	Other	1 Comorbidity	2 Comorbidities	3+ Comordbidities
β (95% CI)	25	7.61 (7.57, 7.63)	0.47 (0.45, 0.48)	0.28 (0.26, 0.29)	0.26 (0.24, 0.27)	0.01 (0, 0.02)	0.03 (0.02, 0.05)	0.11 (0.11, 0.15)
	10	7.58 (7.53, 7.63)	0.46 (0.43, 0.48)	0.28 (0.25, 0.30)	0.26 (0.23, 0.28)	0.0 (-0.01, 0.02)	0.05 (0.03, 0.08)	0.16 (0.13, 0.2)
	5	7.61 (7.54, 7.68)	0.38 (0.35, 0.41)	0.30 (0.26, 0.35)	0.25 (0.21, 0.28)	0.02 (0.0, 0.05)	0.05 (0.02, 0.09)	0.09 (0.05, 0.15)
	1	7.46 (7.32, 7.63)	0.44 (0.37, 0.51)	0.32 (0.22, 0.41)	0.26 (0.2, 0.33)	0.08 (0.03, 0.13)	0.07 (0.02, 0.16)	0.18 (0.08, 0.30)
Random Effects Meta-regression (REMR)								
Parameter	Sample (%)	Intercept	Non-Hispanic Black	Hispanic	Other	1 Comorbidity	2 Comorbidities	3+ Comordbidities
β (95% CI)	100	7.74 (7.58, 7.90)	0.24 (0.07, 0.40)	0.12 (-0.04, 0.28)	0.09 (-0.08, 0.25)	0.10 (-0.06, 0.26)	0.11 (-0.06, 0.27)	0.05 (-0.11, 0.21)

*- Independent variables used in fitting the linear mixed model were: linear time; race (non-Hispanic white reference, indicator variables); sex (female reference); marital status (single reference), service disability percentage, residence status (urban/rural, rural reference), VISN region (Northeast, Mid-Atlantic, South, Midwest, and West), and number of comorbidities (1, 2, or 3+; none reference)

Appendix Table 2. Parameter Estimates*, Standard Errors for binary HbA1c using Simple Random Sampling, Stratified Random Sampling, and Random Effects Meta-regression for Intercept and Race from the General Linear Mixed Model (GLMM†) for Veterans with Type 2 Diabetes (2002-2006) with VISNs 13 and 14

Simple Random Sample (SRS)								
Parameter	Sample (%)	Intercept	Non-Hispanic Black	Hispanic	Other	1 Comorbidity	2 Comorbidities	3+ Comordbidities
β (95% CI)	100‡	-0.94 (-0.98, -0.91)	0.62 (-0.02, -0.01)	0.45 (0.43, 0.48)	0.36 (0.35, 0.38)	0.07 (0.06, 0.08)	0.15 (0.13, 0.17)	0.27 (0.24, 0.29)
	25‡	-1.93 (-2.17, -1.69)	1.27 (1.17, 1.37)	1.07 (0.93, 1.20)	0.87 (0.77, 0.97)	0.12 (0.05, 0.21)	0.19 (0.06, 0.31)	0.39 (0.19, 0.58)
	10‡	-2.48 (-2.93, -2.04)	1.39 (1.20, 1.58)	1.27 (1.01, 1.53)	0.97 (0.78, 1.15)	0.16 (0.01, 0.31)	0.36 (0.14, 0.59)	0.44 (0.07, 0.80)
	5	-2.16 (-2.87, -1.45)	1.69 (1.40, 1.99)	1.10 (0.70, 1.50)	1.05 (0.75, 1.35)	0.39 (0.16, 0.62)	0.34 (0.09, 0.80)	0.33 (-0.24, 0.90)
Stratified Random Sampling (StRS)								
Parameter	Sample (%)	Intercept	Non-Hispanic Black	Hispanic	Other	1 Comorbidity	2 Comorbidities	3+ Comordbidities
β (95% CI)	25	-1.83 (-2.07, -1.59)	1.25 (-0.01, 0.01)	0.90 (0.76, 1.03)	0.88 (0.78, 0.98)	0.10 (0.02, 0.18)	0.30 (0.17, 0.42)	0.75 (0.56, 0.94)
	10	-2.34 (-2.78, -1.89)	1.47 (1.29, 1.66)	1.03 (0.78, 1.29)	1.00 (0.81, 1.19)	0.07 (-0.08, 0.21)	0.24 (0.02, 0.47)	0.69 (0.34, 1.05)
	5	-2.65 (-3.35, -1.95)	1.44 (1.14, 1.74)	1.74 (1.34, 2.15)	1.10 (0.81, 1.40)	0.20 (-0.03, 0.43)	0.16 (-0.19, 0.52)	0.80 (0.22, 1.39)
Random Effects Meta-regression (REMR)								
Parameter	Sample (%)	Intercept	Non-Hispanic Black	Hispanic	Other	1 Comorbidity	2 Comorbidities	3+ Comordbidities
β (95% CI)	100	-0.59 (-0.85, -0.33)	0.16 (-0.10, 0.42)	1.23 (0.97, 1.49)	0.23 (-0.03, 0.49)	0.25 (-0.02, 0.51)	0.27 (0.01, 0.53)	0.50 (0.24, 0.76)

* Independent variables used in fitting the general linear mixed model using a binomial distribution with a logit link function were: linear time; race (non-Hispanic white reference, indicator variables); sex (female reference); marital status (single reference), service disability percentage, residence status (urban/rural, rural reference), and number of comorbidities (1, 2, or 3+; none reference)

Appendix Table 3. Number of subjects by VISN

VISN	*Frequency*	*Percent*
1	39837	4.47
2	24394	2.74
3	38825	4.36
4	57011	6.40
5	21612	2.43
6	44795	5.03
7	54310	6.10
8	90778	10.20
9	42783	4.80
10	34857	3.91
11	41252	4.63
12	38552	4.33
13	2769	0.31
14	2119	0.24
15	41350	4.64
16	78043	8.76
17	42319	4.75
18	37123	4.17
19	20591	2.31
20	26734	3.00
21	32282	3.63
22	40343	4.53
23	37715	4.24

Appendix Table 4. Akaike's Information Criterion (AIC) and Bayesian Information Criterion (BIC) for Continuous and Categorical HbA1c from the Linear Mixed Model (LMM*) and the General Linear Mixed Model (GLMM†) Adjusted for Sample Size for Veterans with Type 2 Diabetes (2002-2006) by type of Approach

Model	Method	Parameter	Full	25%	10%	5%	1%
LMM*	SRS	AIC	7,845,341	11,114,139	13,291,313	14,986,297	18,908,772
		BIC	7,845,570	11,114,945	13,293,163	14,989,752	18,923,189
	Stratified	AIC		11,151,566	13,363,592	14,910,955	18,706,619
		BIC		11,152,193	13,365,032	14,913,641	18,717,820
	Meta-regression	AIC	132,162				
		BIC	132,249				
GLMM†	SRS‡	AIC	11,102,402	11,944,733	12,288,038	12,594,022	13,120,170
		BIC	11,102,580	11,945,350	12,289,463	12,569,653	13,131,149
	Stratified‡	AIC		11,990,004	12,435,629	12,520,621	13,090,347
		BIC		11,990,621	12,437,044	12,523,262	13,101,326
	Meta-regression	AIC	120,146				
		BIC	120,165				

*- Independent variables used in fitting the linear mixed model were: linear time; race (non-Hispanic white reference, indicator variables); sex (female reference); service disability percentage, marital status (single reference), residence status (urban/rural, rural reference), VISN region (Northeast, Mid-Atlantic, South, Midwest, and West, South reference); and number of comorbidities (1, 2, or 3+; none reference)

†- Independent variables used in fitting the general linear mixed model using a binomial distribution with a logit link function were: linear time; race (non-Hispanic white reference, indicator variables); sex (female reference); service disability percentage, marital status (single reference), residence status (urban/rural, rural reference), and number of comorbidities (1, 2, or 3+; none reference)

‡- Could not be fit with R: lmer() Warning Message: "non-integer #successes in a binomial glm!" received. Fit with PROC GLIMMIX and pseudo AIC & BIC are reported

Appendix Table 5. Time (seconds) taken to fit the Linear Mixed Model (LMM*) and General Linear Mixed Model (GLMM*)

Model	Method	Software	Full	25%	10%	5%	1%
LMM	SRS	R	190	29	13	6	1
		SAS	46	14	6	3	1
	REMR	SAS	75				
GLMM	SRS	R	NP				
		SAS	533	98	41	25	4
	REMR	SAS	391				

*- Independent variables used in fitting the models were: linear time; race (non-Hispanic white reference, indicator variables); sex (female reference); service disability percentage, marital status (single reference), residence status (urban/rural, rural reference), VISN region (Northeast, Mid-Atlantic, South, Midwest, and West, South reference); and number of comorbidities (1, 2, or 3+; none reference)

NP- Could not be fit with R: lmer() Warning Message: "non-integer #successes in a binomial glm!" received.

Appendix Table 6. LMM and 95% CI- Simple random sampling (SRS), stratified random sampling (StRS) and random effects meta regression (REMR)

Analysis Variable	SRS 100% (n=890,394		SRS 10% (n=90,000)		StRS 10% (n=90,000		REMR All VISNs		REMR without VISN 13 or 14	
Intercept	7.54	(7.52, 7.55)	7.54	(7.48, 7.58)	7.58	(7.53, 7.63)	7.74	(7.58, 7.90)	7.58	(7.54, 7.62)
Time	0.00	(0.00, 0.00)	0.00	(0.00, 0.01)	0.00	(0.00, 0.00)	0.05	(-0.12, 0.21)	-0.01	(-0.05, 0.03)
Non-Hispanic Black*	0.46	(0.45, 0.46)	0.47	(0.44, 0.48)	0.46	(0.43, 0.48)	0.24	(0.07, 0.40)	0.45	(0.41, 0.49)
Hispanic*	0.29	(0.28, 0.30)	0.30	(0.26, 0.32)	0.28	(0.25, 0.30)	0.12	(-0.04, 0.28)	0.08	(0.04, 0.12)
Other*	0.25	(0.23, 0.25)	0.26	(0.23, 0.27)	0.26	(0.23, 0.28)	0.09	(-0.08, 0.24)	0.23	(0.19, 0.27)
Male†	-0.10	(-0.12, -0.08)	-0.13	(-0.17, -0.08)	-0.10	(-0.16, -0.06)	-0.12	(-0.28, 0.04)	-0.08	(-0.12, -0.04)
Married‡	-0.21	(-0.21, -0.20)	-0.19	(-0.20, -0.17)	-0.21	(-0.22, -0.19	-0.34	(-0.50, -0.18)	-0.21	(-0.25, -0.17)
Disability (%, mean & se) §	0.00	(0.00, 0.00)	0.00	(0.00, 0.00)	0.00	(0.00, 0.00)	0.03	(-0.13, 0..20)	0.00	(-0.04, 0.04)
Northeast‖	-0.03	(-0.04, -0.03)	-0.01	(-0.03, 0.01)	-	-	-	-	-	-
Mid-Atlantic‖	0.06	(0.05, 0.06)	0.06	(0.04, 0.08)	-	-	-	-	-	-
Midwest‖	0.02	(0.01, 0.03)	0.02	(0.00, 0.05)	-	-	-	-	-	-
West‖	0.05	(0.04, 0.06)	0.05	(0.02, 0.07)	-	-	-	-	-	-
Urban Residence¶	-0.02	(-0.02, -0.01)	-0.02	(-0.04, -0.01)	-0.03	(-0.05, -0.02)	0.02	(-0.15, 0.18)	-0.01	(-0.05, 0.04)
1 Comorbidity#	0.01	(0.01, 0.02)	0.03	(0.02, 0.05)	0.00	(-0.01, 0.02)	0.10	(-0.06, 0.26)	0.01	(-0.04, 0.05)
2 Comorbidities#	0.04	(0.04, 0.5)	0.08	(0.07, 0.12)	0.05	(0.03, 0.08)	0.11	(-0.06, 0.27)	0.03	(-0.01, 0.07)
3+ Comorbidities#	0.11	(0.11, 0.13)	0.08	(0.05, 0.13)	0.16	(0.13, 0.20)	0.05	(-0.11, 0.21)	0.09	(0.05, 0.13)

* - Comparison group is to non-Hispanic Whites
† - Comparison group is females
‡ - Comparison groupis Veterans who are single
§ - Service connectedness disability
‖ - Comparison group is the Southern region
¶ - Comparison group is rural residence as define by the Office of Rural Health Rural Urban Commuting Area (RUCA) Codes
# - Comparison group is no comorbidities
\- Not applicable due to sampling by VISN or aggregation by VISN

Appendix Table 7. GLMM and 95% CI-- Simple random sampling (SRS), stratified random sampling (StRS) and random effects meta regression (REMR)

Analysis Variable	SRS 100% (n=890,394	SRS 10% (n=90,000)	StRS 10% (n=90,000	REMR All VISNs	REMR without VISN 13 or 14
Intercept	-0.95 (-0.98, -0.91)	-2.48 (-2.93, -2.04)	-2.34 (-2.78, -1.89)	-0.59 (-0.85, -0.33)	-0.93 (-0.99, -0.87)
Time	-0.02 (-0.03, -0.02)	0.00 (-0.02, 0.02)	0.00 (-0.02, 0.02)	0.01 (-0.25, 0.27)	-0.03 (-0.09, 0.03)
Non-Hispanic Black*	0.62 (0.60, 0.63)	1.39 (1.20, 1.58)	1.47 (1.29, 1.66)	0.16 (-0.10, 0.42)	0.58 (0.52, 0.64)
Hispanic*	0.45 (0.43, 0.47)	1.27 (1.01, 1.53)	1.03 (0.78, 1.29)	1.23 (0.97, 1.50)	0.11 (0.05, 0.17)
Other*	0.36 (0.35, 0.38)	0.97 (0.78, 1.15)	1.00 (0.81, 1.19)	0.23 (-0.03, 0.49)	0.32 (0.26, 0.38)
Male†	-0.10 (-0.14, -0.07)	-0.38 (-0.79, 0.04)	-0.48 (-0.89, -0.07)	-1.09 (-1.35, -0.83)	-0.08 (-0.14, -0.02)
Married‡	-0.33 (-0.34, -0.32)	-0.63 (-0.76, -0.50)	-0.82 (-0.96, -0.69)	-0.70 (-0.96, -0.43)	-0.33 (-0.39, -0.27)
Disability (%, mean & se) §	0.00 (0.00, 0.00)	0.00 (0.00, 0.00)	0.01 (0.00, 0.01)	-0.10 (-0.36, 0.16)	0.00 (-0.06, 0.06)
Urban Residence‖	-0.06 (-0.07, -0.05)	-0.20 (-0.33, -0.07)	-0.21 (-0.34, -0.08)	0.05 (-0.21, 0.31)	-0.02 (-0.08, 0.04)
1 Comorbidity¶	0.07 (0.06, 0.08)	0.16 (0.01, 0.31)	0.07 (-0.08, 0.21)	0.25 (-0.02, 0.51)	0.07 (0.01, 0.13)
2 Comorbidities¶	0.15 (0.13, 0.16)	0.36 (0.14, 0.59)	0.24 (0.02, 0.47)	0.27 (0.10, 0.53)	0.14 (0.08, 0.20)
3+ Comorbidities¶	0.27 (0.24, 0.29)	0.44 (0.07, 0.80)	0.69 (0.34, 1.05)	0.50 (0.24, 0.76)	0.25 (0.20, 0.31)

* - Comparison group is to non-Hispanic Whites
† - Comparison group is females
‡ - Comparison groupis Veterans who are single
§ - Service connectedness disability
‖ - Comparison group is rural residence as define by the Office of Rural Health Rural Urban Commuting Area (RUCA) Codes
¶ - Comparison group is no comorbidities

Appendix Figure 1. LMM Parameter Estimates and Pooled 95% Confidence Bounds for Random Effects Meta-regression (Intercept, Race, comorbidity) with VISNs 13 and 14

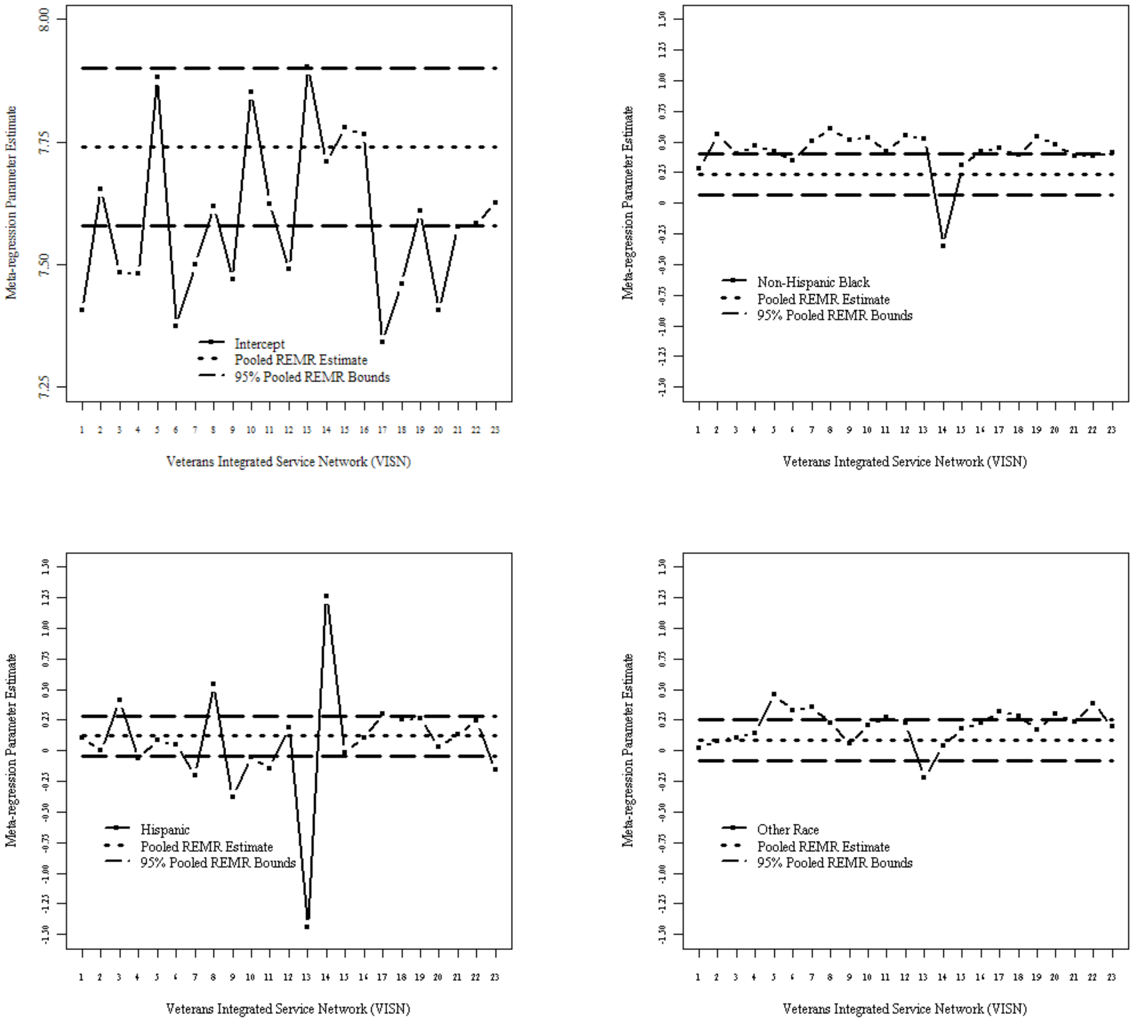

*- Independent variables used in fitting the linear mixed model were: linear time; race (non-Hispanic white reference, indicator variables); sex (female reference); service disability percentage, residence status (urban/rural, rural reference), VISN region (Northeast, Mid-Atlantic, South, Midwest, and West, South reference); and number of comorbidities (1, 2, or 3+; none reference)

Appendix Figure 2. GLMM Parameter Estimates and Pooled 95% Confidence Bounds for Random Effects Meta-regression (Intercept, Race, comorbidity) with VISNs 13 and 14

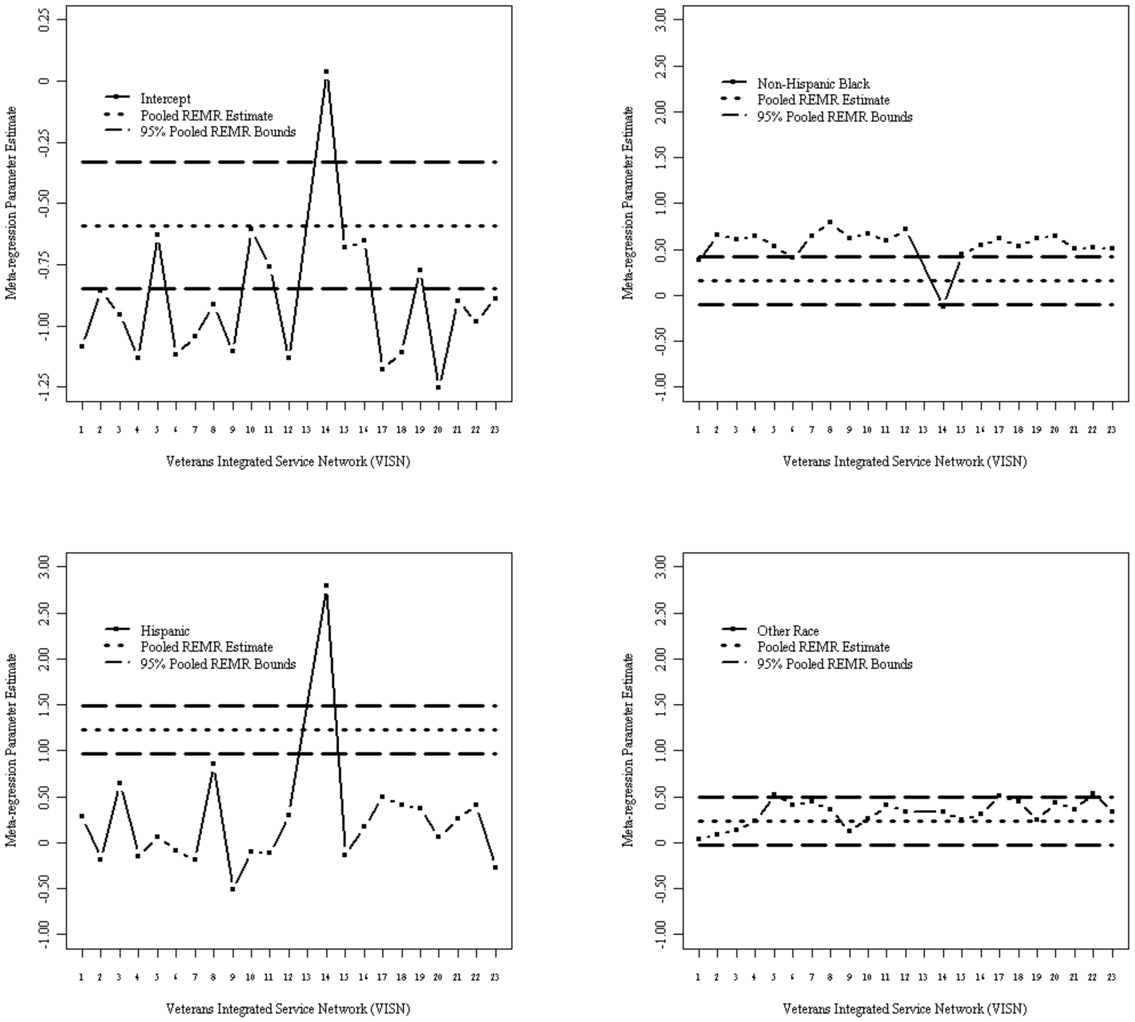

*- Independent variables used in fitting the general linear mixed model using a binomial distribution with a logit link function were: linear time; race (non-Hispanic white reference, indicator variables); sex (female reference); service disability percentage, residence status (urban/rural, rural reference), and number of comorbidities (1, 2, or 3+; none reference)
